# Mapping the Spatial–Temporal Distribution and Migration Patterns of Men Who Have Sex with Men in Mainland China: A Web-Based Study

**DOI:** 10.3390/ijerph17051469

**Published:** 2020-02-25

**Authors:** Dacang Huang, Jinfeng Wang, Tengfei Yang

**Affiliations:** 1 State Key Laboratory of Resources and Environmental Information System, Institute of Geographic Science and Natural Resources Research, Chinese Academy of Sciences, Beijing 100101, China; huangdc@lreis.ac.cn; 2 University of Chinese Academy of Sciences, Beijing 100049, China; yangtf@radi.ac.cn; 3 Institute of Remote Sensing and Digital Earth, Chinese Academy of Sciences, Beijing 10094, China; 4 Aerospace Information Research Institute, Chinese Academy of Sciences, Beijing 10094, China

**Keywords:** men who have sex with men, distribution, migration, China, HIV/AIDS

## Abstract

The human immunodeficiency virus (HIV) infection rate for men who have sex with men (MSM) has rapidly increased in recent years in China and the migrant population accounts for a large proportion of this increase. The migration of MSM not only poses difficulties for government departments charged with treating the disease, but also increases the spread of HIV in geographical space, so it is important to understand the geographical distribution and migrant patterns of MSM. We searched the largest dating website in China to obtain open information from all users in the Chinese mainland from January 2006 to August 2017. For the analysis, the datasets were merged according to units of time and administrative regions. In total, 1,356,609 records were obtained for this study. The main users of the website were single males aged 18–35 years old. Most of the users were located in the large and mid-sized cities of East China. The distribution of MSM was strongly associated with the distribution of the development of service industry in geographical space. The main flow of MSM are mainly located inside the province as internal flow. For those MSM who prefer to migrate to other provinces, the Beijing-Tianjin-Hebei area, the Yangtze River Delta, the Pearl River Delta, and Sichuan and Chongqing area were their primary destinations. The interprovincial migration behavior of MSM was closely related to an increased average income. MSM prefer to migrate to cities with developed economies and open cultures. It is important to strengthen the management of migrant MSM and increase their basic understanding of HIV.

## 1. Introduction

The transmission of the human immunodeficiency virus (HIV) has been increasing significantly among men who have sex with men (MSM). Because HIV can spread quickly in the social cycle of MSM [1], this demographic has become a global burden in relation to the prevalence of HIV [2]. China has a large population of MSM, with previous research estimating that there are between 3,100,000 and 6,300,000 MSM in the country [3]. The infection rate of HIV among MSM in China increased slightly from 2.5% in 2006 to 5.7% in 2010, and sharply increased from 7.5% in 2013 to 25.5% in 2017 [4,5,6]. Among the self-reported cases of MSM who were infected with HIV in 2008–2015, the migrant population accounted for 46.1% [5]. Migrant MSM have become a major group who are vulnerable to HIV.

The migration of MSM influences the size estimation of MSM and local HIV care services, and may also result in the spread of the HIV in geographical areas [7]. Previous studies have shown that MSM who live in places with high AIDS epidemics, such as Southwest China, tend to migrate to East China [7,8]. The geographical spread of the virus may also cause it to move from MSM who are at high risk to the general population. Therefore, it is important to understand the geographical distribution and migrant patterns of MSM.

As an important part of China’s labor force, hundreds and thousands of people in rural areas leave their hometowns to work in cities. According to the survey and monitoring report of migrant workers in 2018 and released by the National Bureau of Statistics (NBS) of China, 288 million migrant workers migrated from underdeveloped areas to developed areas of China in 2018, and males accounted for 65.2% of this figure [9]. Coupled with the sizable male population who study and work in the big cities, the size of the floating male population is large.

MSM are typically part of this floating male population, and their migration is affected by numerous factors. The uneven development of urban and rural areas, increased job opportunities, better educational environments and the openness and tolerance of big cities, etc., have attracted more gay men to leave their hometowns and gather in these cities [10,11]. MSM from rural areas and small cities tend to move to bigger cities with larger and more mature communities.

The characteristics of migrant MSM make them vulnerable to HIV. Most of the migrant population of MSM are young or middle-aged and sexually active. Living alone and being tempted by urban living environments makes them inclined to seek out sexual stimulation [12]. In addition, most MSM lack knowledge of safe sex, do not use condoms and have multiple sexual partners and sexual roles [13,14]. These factors all increase their risk of HIV infection [12,15].

Under the pressure of the traditional culture of China as in other countries, MSM seek to hide their sexual behavior. They tend to seek out sex using online dating tools such as dating forums, dating websites and geosocial networking apps. These tools provide researchers with the potential to study MSM and HIV around the world. For example, geosocial networking apps have been used to calculate the local population density of MSM and determine the high-density areas of minority and young-minority MSM in Atlanta (GA, USA) [16]. The relationship between the use of geosocial networking apps and the presence of business zoning and population density was examined in a mid-sized city in southern states of the US [17]. The online profiles of MSM were used to explore geographical variations in the sexual preferences and expectations of MSM across 15 selected cities in the US. The findings showed that the use of condoms and the status of reported HIV differed significantly in these cities [18].

In 2016, Mi et al. used the addresses of users’ profiles collected from BF99—the largest dating website in China—to explore the migration patterns of MSM in China [7]. The study emphasized an analysis of the migration routes of MSM from southwestern China to other parts of China. However, a visualization of the migration patterns of MSM throughout China and an analysis of other areas of China are still needed. In this study, we searched the largest dating website of China to collect the open data of MSM and conduct a visualization and analysis of the spatial–temporal distribution and migrant patterns of MSM in China. We also explored the potential effects that economic factors have on the spatial distribution and migration of MSM.

## 2. Materials and Methods

### 2.1. Data Sources

The BF99 dating website (http://www.bf99.com/) was established in 2000 when Internet usage became popular in Mainland China. It is the largest dating website for gay men in Mainland China and has users in more than 10 countries and regions. By June 7, 2019, the website already had 2,946,880 registered users. Most of the users were located in large and mid-sized cities in China [19].

When users register on the website, they are required to provide photos and personal information to create their profile; this helps other users get to know them. The personal information includes their sex, age, occupation, personal interests, etc. Users can choose to have some of their information open to the public—this allows for more convenient interactions with other users. This information can be seen by anyone, as individuals do not need to log on to the website to see it. Other important personal information, such as personal contact methods, can only be seen by logging on to the website.

We searched the website to obtain open information about all users in Mainland China from January 2006 to August 2017. After eliminating the information that did not have any significant attributes, 1,356,609 records were obtained. The relevant attributes included each user’s nickname, age, height, weight, occupation, marital status, current location, birthplace, dating purpose, sexual role preference, relationship status and attitude toward marriage. We merged the datasets according to units of time and administrative regions. No personal information was included in the study.

The economic level and development status of different cities influence the geographic mobility of MSM and consequently form the spatial patterns of the distribution of MSM. Because gross domestic product (GDP) and industrial structure represent the economic level and development status of a city, they were used to represent the economic factors in this study. The NBS classifies the industries of China into three groups. The first industry includes agriculture, forestry, animal husbandry and fishery (excluding services of these four industries); the second industry includes mining (excluding mining-support activities), manufacturing (excluding metal products, machinery and equipment repair), electricity, heat, gas, water production and supply, and construction; the third industry is the service industry, which refers to industries other than those in the first and second industries [20]. We collected the GDP and industry data for 2010 from the official website of the NBS.

MSM who leave their hometowns for a new location form a population flow in geographic space. In order to observe this phenomenon in geographic space, the flow of MSM was counted using the administrative units at province and city level. The migration of MSM in geographical space is affected by different factors, such as cultural, economic, familial and societal factors, etc. In this study, we focus on quantifying the impact of spatial distance, unemployment rates and income on the flow of MSM in geographic space. In order to model this effect, the spatial distance, male population aged 15–69 years, the unemployment rate in the source area (i.e., the hometowns of each MSM) and the average income of urban residents in the sink area (i.e., the location of each MSM) were chosen as explanatory variables for modeling. Among them, the data of males aged 15–69 years were derived from the national census data of 2010; the unemployment rate and average income of urban residents were obtained from the 2010 China City Statistical Yearbook.

### 2.2. Statistical Analysis

In order to quantify the effects of economic factors on the spatial distribution of the MSM population, the geodetector [21] method was used to analyze the relationship between the number of MSM users and the economic factors in geographical space.

The geodetector model is as follows:

(1) $$q=1-\frac{1}{ℜ\sigma^{2}}\sum_{h=1}^{L}{ℜ}_{h}\sigma_{h}^{2}$$

where q∈[0,1]; $\sigma^{2}=\frac{1}{n}{\sum_{i=1}^{n}\left(y_{i}-\bar{y}\right)}^{2}$ represents the variance in a population that contains n samples ($y_{i}$) with a mean value of $\bar{y}$; $\sigma_{h}^{2}=\frac{1}{n_{h}}{\sum_{i=1}^{n_{h}}\left(y_{hi}-{\bar{y}}_{h}\right)}^{2}$ indicates the variance in each subpartition; ℜ is the number of elements of the population; ${ℜ}_{h}$ represents the number of elements in subpartition h; and L is the number of partitions in the population. The large value of q indicates that this factor had a greater effect on the spatial distribution of MSM in this research [21,22]. Detailed instructions for using the geodetector method and software can be found online (http://www.geodetector.cn/).

The classic model for flow data is the gravity model [23,24]. In Equation (2), $T_{ij}$ represents the flow between region $V_{i}$ and region $W_{j}$; $V_{i}^{u}$ refers to the variables of the source area; $W_{j}^{a}$ represents the variables of the sink area; $d_{ij}^{\beta }$ refers to the spatial distance between region $V_{i}$ and region $W_{j}$; k indicates coefficients; i, j are integers; u, α and β are the coefficients of V, W and d [24]:

(2) $$T_{ij}=k\frac{V_{i}^{u}W_{j}^{a}}{d_{ij}^{\beta }}$$

By expanding the gravity model in the logarithm, Equation (3) can be obtained:

(3) $$\ln T_{ij}=k+u\ln V_{i}+\alpha \ln W_{j}-\beta \ln d_{ij}$$

By putting the variables of the male population aged 15–69 years and the unemployment rate in the source area, the average income of urban residents in the sink area and the spatial distance into Equation (3), Equation (4) can be obtained. Then, a Poisson regression model can be used to estimate the coefficients and improve the accuracy of the coefficient estimation:

(4) $$\begin{array}{l} \ln{\left(\mathrm{Flow}\right)}_{ij}=k+u_{1}\ln{\left(\mathrm{MalePopulation}\right)}_{i}+u_{2}\ln{\left(\mathrm{UnemploymentRate}\right)}_{i}\\ +\alpha \ln{\left(\mathrm{AverageIncome}\right)}_{j}-\beta \ln{\left(\mathrm{Distance}\right)}_{ij} \end{array}$$

## 3. Results

### 3.1. Sociodemographic Characteristics of the Population of Men Who Have Sex with Men (MSM) on BF99

Table 1 presents the sociodemographic characteristics of MSM users on BF99. The average age of gay users was 31 years; the minimum age was 17 years; the maximum age was 68 years; and the median age was 29 years. Users who were 18–35 years old accounted for 79.2% of all users and comprised the main users of the website.

Students and those who were self-employed accounted for 31.75% of all occupations. Six other occupations, including that of engineer, service worker, IT technician, head of an institution, company manager and salesperson accounted for 20.3% of all users. In total, these eight occupations comprised 52.05% of all occupations.

The main users of the website were unmarried. However, 8.13% of users had chosen to marry a heterosexual woman, 4.03% had chosen to marry a lesbian and 4.02% were divorced.

### 3.2. Spatial-Temporal Distribution of MSM Users

**Figure 1:**
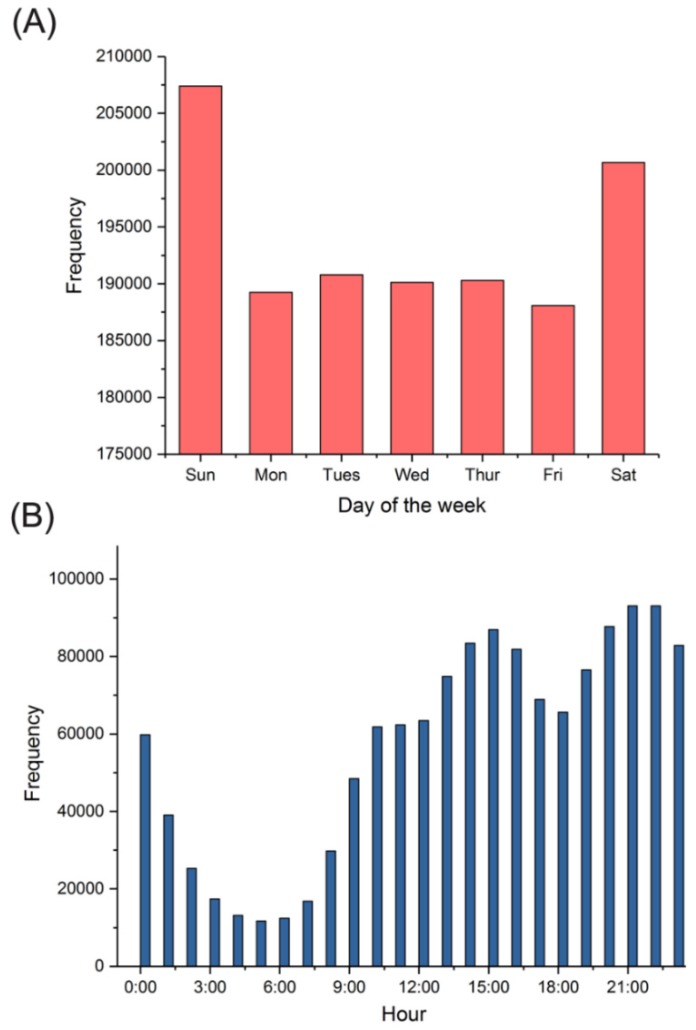
The frequencies of log-on times for men who have sex with men (MSM) users on BF99. (**A**) Frequency statistics of days of the week. (**B**) Frequencies statistics of hours of the day.

By analyzing the last log-on time of the gay users on the website, we found that MSM tended to visit the website on Saturdays or Sundays and that 14:00–16:00 and 20:00–23:00 were peak visiting hours (Figure 1). These results indicated that MSM preferred to search for new partners online in the afternoon or at night, especially at weekends.

**Figure 2:**
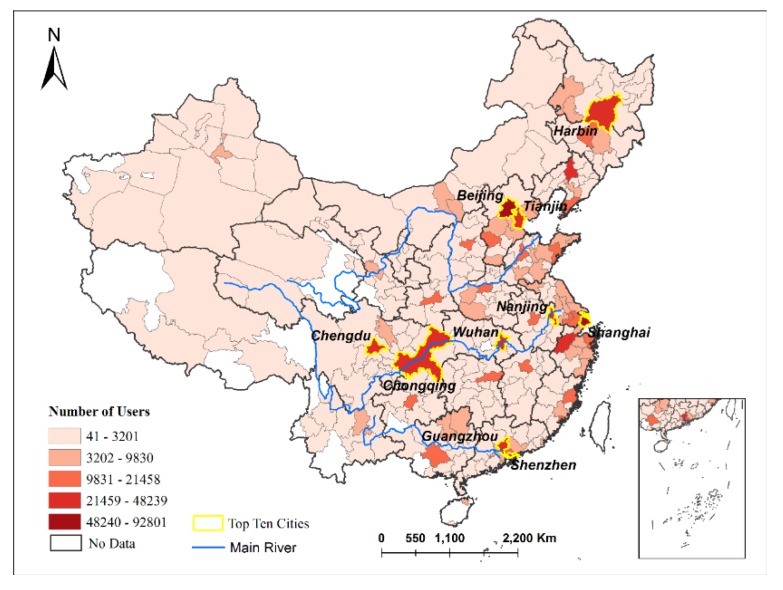
The spatial distribution of MSM users of BF99. Cities that have more users are highlighted with yellow border.

By analyzing the log-on locations of MSM users, we found that most users were in the large and mid-sized cities of East China (Figure 2). These cities have developed economies, provide many jobs and have relatively open cultures.

### 3.3. The Migration Patterns of MSM

**Figure 3:**
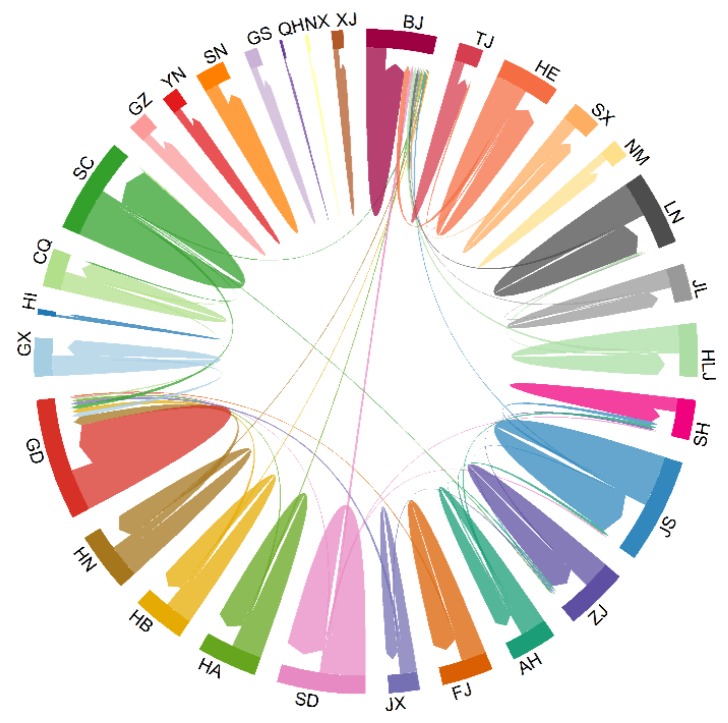
Overall migration patterns of provincial population flows. The main flow of MSM was predominantly located within the province as internal flow. Abbreviations: BJ, Beijing; TJ, Tianjing; HE, Hebei province; SX, Shanxi province; NM, Inner Mongoria; LN, Liaoning province; JL, Jilin province; HLJ, Heilongjiang province; SH, Shanghai; JS, Jiangsu province; ZJ, Zhejiang province; AH, Anhui province; FJ, Fujian province; JX, Jiangxi province; SD, Shangdong province; HA, Henan province; HB, Hubei province; HN, Hunan province; GD, Guangdong province; GX, Guangxi province; HI, Hainan province; CQ, Chongqing; SC, Sichuang province; GZ, Guizhou province; YN, Yunnan province; XZ, Tibet; SN, Shaanxi province; GS, Gansu province; QH, Qinghai province; NX, Ningxia; XJ, Xinjiang.

The migration patterns of MSM on BF99 were first assessed at the provincial level by measuring the population flow from one province to another. Several phenomena were observed. First, due to the restriction of the geographical distance, we found that the main flow of MSM was mainly located inside the province as internal flow (Figure 3). The largest MSM populations were found in Guangdong, Jiangsu, Beijing, Sichuan and Shandong. The internal flow within the province was much larger than the inter-provincial flow.

**Figure 4:**
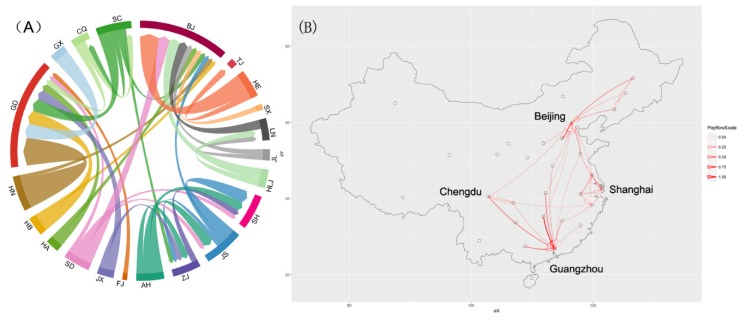
The migration patterns of MSM with an inter-provincial population flow above 2000. (**A**) Chord diagram. (**B**) Geographical distribution.

Second, from the perspective of the outflow of the provincial population, the gay population mainly flowed from provinces across the country to the Beijing-Tianjin-Hebei area, the Yangtze River Delta, the Pearl River Delta, and Sichuan and Chongqing area, thus forming a diamond-shaped structure (Figure 4). The Beijing-Tianjin-Hebei area, the Yangtze River Delta and the Pearl River Delta are three major urban agglomerations and industrial areas in China, while Sichuan and Chongqing are new economic growth poles of inland China and have large populations. They all have the resources to attract MSM.

### 3.4. Relationship between the Distribution of MSM and GDP

**Figure 5:**
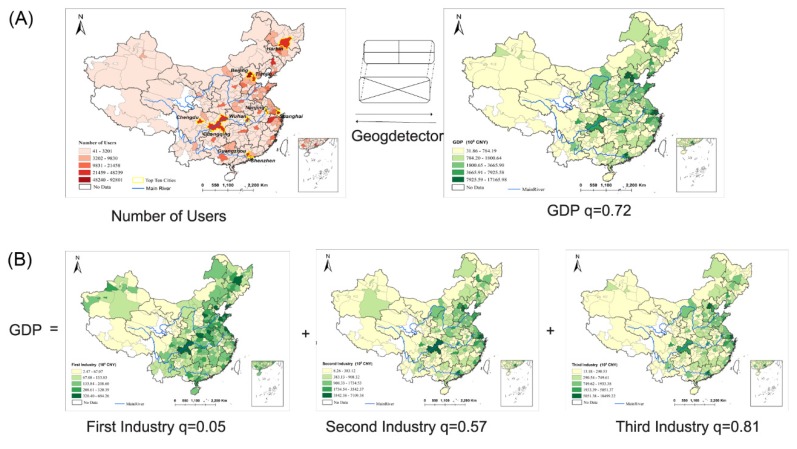
Relationship between the distribution of gay users and GDP, three groups of industries. (**A**) Relationship between the distribution of gay users and GDP; (**B**) Relationship between the distribution of gay users and three groups of industries. The strongest relationship was observed between the distribution of MSM users and the third industry.

The geodetector method was used to analyze the relationship between the distribution of the number of MSM users and certain economic factors. The natural breaks method was used to classify the GDP, the first industry, the second industry and the third industry into five levels using ArcMap software. They were then compared with the distribution of MSM users using the geodetector method. The q value of GDP was 0.72. However, after separating GDP into three group of industries, we found that the q values of the first, second and third industries were 0.05, 0.57 and 0.81, respectively (Figure 5). The first industry had a weak relationship with the distribution of MSM users in geographical space, and the second industry had a medium relationship. The strongest relationship was observed between the distribution of MSM users and the third industry. This result indicates that cities with a developed third industry are more attractive to gay men than other cities are. Cities with a developed third industry also have a developed service industry, provide more jobs and have open cultures. These factors may induce gay men to migrate to these cities.

### 3.5. Modeling the Impact of Spatial Distance and Income on MSM Flow

To determine the impact of spatial distance and income on MSM flow, the variables of spatial distance, the male population aged 15–69 years in the source area, the unemployment rate in the source area and the average income of urban residents in the sink area were put into Equation (4).

The modeling results (Table 2) show that the longer the spatial distance, the smaller the flow from source area to sink area. The results also show that the larger the male population at the source, the higher the unemployment rate at the source, and the higher the average income at the sink, the more MSM flow from source area to sink area. The average income in the sink area had the largest impact on the increment of MSM flow, thus showing that the migration of MSM in geographic space is related to the pursuit of higher income.

## 4. Discussion

Homosexuality has been recorded in China’s history since ancient times [25,26,27]. Dating back to the last century, homosexuality was still regarded as a psychiatric disorder, and homosexual activity was treated as hooliganism [28]. Because homosexuality was not socially acceptable, most gay people could only express their yearning and love for others in a concealed way [29], and they typically met each other in toilets, bathhouses and parks [30,31,32]. It was not until 1997 that the criminal law in China ended the penalty for homosexual behavior [33]. Thanks to the rapid development of the Internet, MSM began to use various web tools to communicate with each other [34,35,36,37]. These new tools have created opportunities for the monitoring of MSM. In this study, we used the open data collected from the largest dating website of MSM in China to visualize and analyze the spatial–temporal distribution and migrant patterns of MSM in China and explore the potential effects of economic factors on the spatial distribution and migration of MSM.

The sociodemographic characteristics of MSM on the website showed that most MSM in the study sample were young and single. Users who were 18–35 years old were the main users of the website. Most MSM could not find lovers on the Internet or establish stable relationships with others [38]. Gay marriage is still socially unacceptable in China [39,40]. Some gay men have to marry heterosexual women because of the pressures of their parents and public opinion; this leads to the phenomenon of a homosexual man having a heterosexual spouse. In our study sample, 8.13% of MSM chose to marry a heterosexual woman. It was estimated that about 16 million heterosexual spouses are married to gay men in China [41], and most of these spouses did not know that their partner was gay before they married.

The log-on times and locations show the spatial–temporal distribution patterns of MSM on this website and can help us better understand them. First, regarding the log-on times of the website, it was found that MSM like to visit the website at weekends and that 14:00–16:00 and 20:00–23:00 were the peak visiting hours. Therefore, it could be more effective to spread prevention knowledge regarding HIV/AIDS during this period, as it would have improved reach. Second, regarding the locations of MSM users, we found that most users were in the large and mid-sized cities of East China. The distribution of MSM users had a strong relationship with the third industry. Cities with a developed third industry may create more opportunities and open cultures for MSM and induced them to gather in these cities. Thus, it is important to strengthen the monitoring of MSM in cities with a developed third industry.

MSM who leave their hometowns for a new location form population flow in geographic space. In exploring the migration patterns of MSM in China, the study found that the main flow of MSM is mainly located inside the province as internal flow. This may be due to the restrictions of geographical distance. As well as this, most Chinese citizens are fond of their hometowns and prefer to study and find jobs near their hometowns. For those MSM who prefer to migrate to other provinces, the Beijing-Tianjin-Hebei area, the Yangtze River Delta, the Pearl River Delta, and Sichuan and Chongqing area were their primary destinations. The outflow of MSM in all provinces formed a diamond-shaped structure in space. These four areas have their own advantages and currently represent the most developed regions of China. By modeling the relationship between MSM flow and geographical distance, the unemployment rate, average income and male population aged 15–69 years, we find that the interprovincial migration behavior of MSM is closely related to increased average income. The effect of spatial distance on the outflow of MSM (−0.91) was less than the effect of average income on MSM (+2.11).

This research has some limitations. First, because some MSM may not use the Internet to find their partners, the characteristics of sex-seeking gay men on the website examined in this study may be different from the wider population of gay men in China. In this study, we found that MSM who used the dating website tended to be younger than the general population. Students and single men were the main users. Zou et al. also found that compared with MSM who do not use mobile apps, MSM who used mobile apps tended to be younger, have higher education levels and incomes, and are more likely to partake in risky sex behavior [42]. Second, the migration of MSM may be affected by different factors, such as cultural, economic, familial and societal factors, etc. Constrained by the attributes of the data recorded by the website, many individual attributes cannot be obtained for a deeper analysis. The study mainly focused on quantifying the impact of spatial distance and economic factors on the influence of MSM flow in geographic space from a regional perspective.

To reduce the infection rate of HIV among migrant MSM, some methods can be considered for adoption in the future. First, an information management system of migrant MSM should be established. Health departments should conduct a detailed survey of the working and living conditions of migrant MSM and provide them with necessary assistance. Second, relevant regulations should be completed to protect their legitimate rights and interests. Third, because the sexual behavior of MSM is more dangerous of affecting HIV than the sexual behavior of heterosexuals [43], medical departments should improve their medical services, strengthen health promotion and safe-sex education, and improve basic understandings of HIV among MSM. Fourth, we can mobilize gay men to carry out HIV testing with the help of their peers and let them know the benefits of HIV testing in terms of their physical and mental health. Fifth, outreach activities can be carried out regularly at common dating places for MSM. This can encourage MSM to undergo HIV testing and make the public more aware of their living conditions. Sixth, psychological counselling services should be provided to MSM to help them solve emotional problems, marital problems, etc. All of these may help reduce the infection rate of HIV among MSM.

## 5. Conclusions

Migrant MSM have become a major demographic who are vulnerable to HIV in China [5]. By exploring open data from the largest dating website in China, we found that the main users of MSM were located in the large and mid-sized cities of East China. The distribution of MSM had a strong relationship with the development of the third industry. The main flow of MSM is mainly located within the province as internal flow. For those MSM who prefer to migrate to other provinces, the Beijing-Tianjin-Hebei area, the Yangtze River Delta, the Pearl River Delta, and Sichuan and Chongqing area were their primary destinations. The interprovincial migration behavior of MSM is closely related to increased average income. It is important to strengthen the management of migrant MSM, increase their basic understanding of HIV, mobilize them to get tested for HIV and reduce the prevalence of HIV among them.

## Figures and Tables

**Table 1:** Sociodemographic characteristics of MSM population on BF99 in China, 2006–2017.

Category	Number of Users	Percentage
**Age (Median: 29 Years)**		
Under 18 years	4951	0.36
18–25	279,857	20.63
26–30	500,195	36.87
31–35	294,348	21.7
36–40	130,386	9.61
41–50	112,422	8.29
61–60	25,121	1.85
Above 60 years	9329	0.69
**Occupation**		
Student	275,150	20.91
Self-employed	142,581	10.84
Engineer	55,061	4.19
Service worker	52,734	4.01
IT technician	44,782	3.4
Head of an institution	39,329	2.99
Company manager	38,659	2.94
Salesperson	36,456	2.77
Other	630,900	47.95
**Marital Status**		
Single	1,136,982	83.81
Married to a heterosexual woman	110,332	8.13
Married to a lesbian	54,716	4.03
Divorced	54,566	4.02

**Table 2:** Coefficients of the variables of the spatial interaction model.

Variables	Coefficients	Standard Deviation	Z Value	Pr (>|z|)
Intercept	5.83	0.02	236.32	<0.01
Distance	−0.91	0.002	−406.21	<0.01
Male population aged 15–69 years (source area)	0.63	0.003	242.42	<0.01
Unemployment rate (source area)	0.49	0.009	55.12	<0.01
Average income (sink area)	2.11	0.005	409.83	<0.01

Note: The Z value is computed as the test statistic for the hypothesis test that the true corresponding regression coefficient is 0. Pr means probability.
